# Quasi-experimental design for using an interactive social media intervention program to improve truck drivers’ health beliefs and eating behaviors

**DOI:** 10.1186/s12889-022-13883-6

**Published:** 2022-08-04

**Authors:** Ssu-Lan Chang, Wen-Chi Wu, Yih-Jin Hu, Hsin-Yi Lai, Te-Chih Wong

**Affiliations:** 1 Department of Health Promotion and Health Education, College of Education, National Taiwan Normal University, No. 162, Section 1, Heping East Road, 10610 Taipei, Taiwan; 2 Department of Nutrition, Cathay General Hospital, 280 Renai Rd. Sec.4, Taipei, Taiwan; 3 Department of Internal Medicine, National Taiwan University Hospital, 7, Chung Shan S. Rd, Zhongzheng Dist, Taipei, Taiwan; 4 Department of Nutrition and Health Sciences, Chinese Culture University, Yang-Ming-Shan, 55, Hwa-Kang Rd, Taipei, Taiwan

**Keywords:** Social media intervention, Quasi-experimental design, Truck driver, Health Belief Model

## Abstract

**Background:**

Truck drivers have difficulties participating in health education programs delivered at a fixed time and place due to the mobility of their workplace. Interventions conducted via social media can overcome these limitations of time and place. This study aimed to investigate the effect of a nutrition education intervention program delivered via a social media platform on the healthy eating behaviors of truck drivers.

**Methods:**

This study adopted a quasi-experimental design. A 12-week intervention program was conducted for a social-media group (*n* = 125) and a conventional-teaching group (*n* = 117) from February to May 2020. The social-media group participated in a social-media-based health intervention on the LINE application. The intervention involved the provision of online messages, online instant responses, a picture-based food log, an audio e-book, and a loyalty e-card. The conventional-teaching group participated in a healthy diet course and a hygiene education manual. The generalized estimation equation (GEE) was applied to evaluate the intervention effects on the outcome measures derived from the Health Belief Model.

**Results:**

The results of the GEE showed the social-media-based intervention strategies significantly decreased perceived barriers of consuming a healthy diet (*p* =  < 0.001), increased willingness to follow cues of action (*p* = 0.036), improved the self-efficacy of healthy eating behaviors (*p* = 0.001), and increased the score of healthy eating behaviors (*p* < 0.001) compared with the conventional teaching strategies. For the social-media and conventional-teaching groups, no significant changes occurred in self-perceived health status, self-perceived susceptibility, or self-perceived severity after the intervention. More than 90% of the participants in the social-media group believed the social-media-based intervention strategies could help implement and maintain healthy eating behaviors.

**Conclusions:**

The results indicate social-media-based intervention strategies can facilitate approaching a population without a fixed workplace, such as truck drivers. Health promoters and planners focusing on occupational health can consider developing social-media-based intervention strategies for improving truck drivers' health status.

**Supplementary Information:**

The online version contains supplementary material available at 10.1186/s12889-022-13883-6.

## Introduction

Truck drivers play an important role in countries worldwide because the established logistics network underpins the economic development of many countries. Approximately 2 million long-distance truck drivers work in the United States [1]. Their obesity rate is twice that of people from other occupations, and their metabolic syndrome morbidity (i.e., having more than three of these symptoms: glucose intolerance, low HDL cholesterol, elevated triglycerides, hypertension, and central obesity) is 2.5 higher than that of the general US population [2, 3]. In Taiwan, there are approximately 400,000 truck drivers [4]. However, no research has reported the prevalence of chronic diseases among Taiwanese truck drivers. Only one study has examined the health of Taiwanese bus drivers. This study found more than 50% of bus drivers were overweight [body mass index (BMI) ≥ 25)] [5]. Given that the working situation of bus drivers resembles that of truck drivers, the health of Taiwanese truck drivers warrants further investigation.

For adults, poor dietary habit is one of the major risk factors for metabolic syndrome [6, 7]. Studies about truck drivers have revealed an unbalanced diet and excessive intake of high-fat foods are the primary factors affecting their health [8–11]. Therefore, an intervention program was implemented in the present study to improve the healthy eating behaviors of Taiwanese truck drivers.

Interventional studies have shown diet adjustment as an intervention strategy can effectively mitigate health problems caused by being overweight and metabolic syndrome in adults [12, 13]. The dietary intervention programs used in these studies commonly included personalized dietary advice [14], self-management and monitoring [15, 16], one-on-one consultation [14, 17], group courses [18], telephone follow-up [19], and the provision of a healthy dietary environment [20, 21]. However, because of irregular break times, mainly due to traffic conditions or delivery schedules, truck drivers face difficulty eating a healthy diet or accessing advice at a certain location. Thus, few truck drivers participated in health promotion programs conducted in previous studies employing a control group, where the average number of participants in each group ranged between 20 and 40 people [16, 22–25]. Accordingly, an intervention program not restricted by time and location should effectively increase the participation of truck drivers in health promotion programs.

Because of the ubiquity of the Internet and mobile phones, the use of social media for interventional research has many advantages. First, participants are less restricted by time and space and can easily obtain health information using social media for intervention programs. Conventional intervention methods require drivers to meet up [26, 27]. Secondly, the anonymity of social media increases the willingness of participants to discuss sensitive topics and complex messages with health professionals [28]. Thirdly, compared with conventional intervention programs, social-media-based intervention programs have lower economic cost and higher ubiquity [29, 30]. Finally, because of the advantage of real-time feedback on social media, social-media-based interventions can improve the participants’ compliance [31]. Past studies have used social media to design intervention programs and have effectively reduced adults’ smoking behavior [31] and weight [32] as well as improving the quality of life of diabetics [33]. However, social media have not been applied to conduct an intervention related to the healthy eating behaviors of truck drivers in Taiwan.

Studies have used the Health Belief Model (HBM) to develop different nutrition education and intervention programs for patients with diabetes, university faculty, and female university students [34–38]. The results of these studies indicated the programs can effectively improve participants’ biochemical status, enhance their knowledge, increase their self-efficacy, and promote healthy eating behaviors. Thus, this study adopted the HBM as a framework for developing intervention programs. A quasi-experimental design was adopted for using social media as a platform to conduct an intervention program to improve the health beliefs and promote the healthy eating behaviors of Taiwanese truck drivers. We used LINE (the most popular communication app in Taiwan) to establish an online community for truck drivers, and conducted a 12-week intervention program. This study aimed to investigate the effectiveness of the social media intervention program on the perceived susceptibility, perceived severity, benefits, barriers, and self-efficacy of healthy eating behaviors among Taiwanese truck drivers.

## Methods

### Study design

**Fig. 1:**
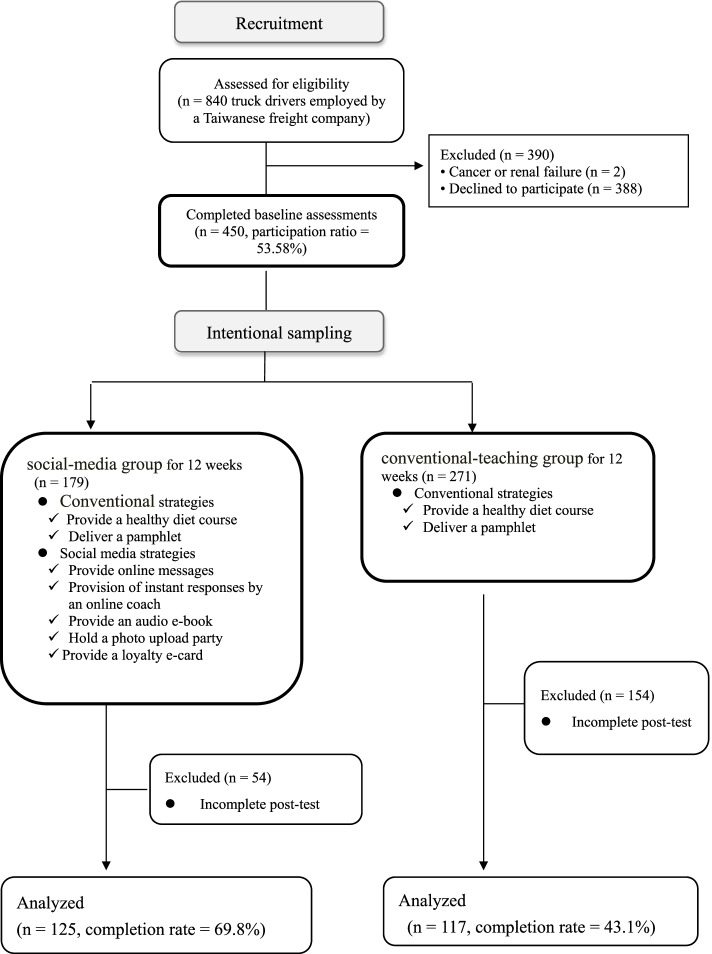
Study flow chart

This study recruited truck drivers from a Taiwanese freight company. Of the 840 truck drivers in this company, 450 agreed to participate in the present study. Since the participants could decide whether to join the experimental group, it was not feasible methodologically to enforce random assignment. Thus, we adopted a quasi-experimental research design (Fig. 1) with 179 participants as the experimental group and 271 participants as the control group [39]. The participation rate was 53.6%. A total of 125 experimental group participants and 117 control group participants completed the pretest and post-test. The data of these 242 participants were included in the final statistical analysis. A goodness-of-fit test on the final and initial analysis samples indicated no significant difference in these samples according to educational levels, marital status, diagnosis with metabolic syndrome, body type, age, BMI, self-perceived health status, self-perceived susceptibility, self-perceived severity, perceived barriers, cues to action, self-efficacy, or healthy eating behaviors (*p* > 0.05).

The control group participated in a conventional health program, which involved a healthy diet course and the use of a nutritional education manual for intervention programs. The experimental group participated in a social-media-based health program which involved conducting health interventions through social media in addition to classroom teaching and manual use. The social media application used in this study was LINE, which is used by 91.3% of the population in Taiwan [40]. The functions of the LINE Official Account include scheduled push notifications, one-to-one real-time feedback, a built-in loyalty e-card, and automatic question-and-answer responses. A licensed dietitian and a trained nurse administered the LINE group for enhancing, teaching, and reinforcing healthy eating behaviors. They both received standardized training at the principal investigator’s office to ensure the consistency of their responses.

In October 2019, we conducted a focus group discussion with the freight company’s manager, workplace nurses, and employee representatives to jointly develop intervention approaches and incentive programs. Subsequently, participants were recruited in January 2020. They were required to sign a consent form and were asked to fill in a pretest questionnaire before joining the social media group (experimental group) or conventional course group (control group) according to their personal preference. A 12-week intervention program was conducted from February to April 2020. After the intervention, the participants were asked to fill in a post-test questionnaire in May 2020. The research tools, research process, and informed consents were reviewed and approved by the Institutional Review Board of Cathay General Hospital (CGH-P108115).

### Participants

We posted recruitment advertisements at truck driver rest areas in the freight company’s five branches in northern, central, and southern Taiwan. In January 2020, the principal investigator visited each branch to explain this research to the company’s drivers. The exclusion criteria of this study were as follows: (1) those without medical examination data, (2) those with renal insufficiency (patients who had been diagnosed with renal insufficiency by doctors, regardless of the stage), (3) those who had been diagnosed with cancer and did not receive treatment or were still under treatment, (4) those with unstable cardiovascular disease (those who had been hospitalized for related diseases or who had had their medication adjusted within 1 month), and (5) those who did not own a smart phone. Those who agreed to participate in the social media group were asked to scan a QR code with their mobile phone to join a LINE group to facilitate the intervention program. Participants who completed the pretest and post-test questionnaires received a gift worth US$13 after completing the post-test.

### Intervention strategies

In this study, an intervention program was designed based on five concepts of the HBM: self-perceived severity, self-perceived susceptibility, perceived barriers, self-efficacy, and cues to action. The purpose of this program was to improve participants’ awareness of disease risks, improve their self-efficacy and perceived benefits, reduce their perceived barriers, and promote changes in their healthy eating behaviors by increasing their exposure to cues to action. The detailed content of the intervention program is shown in the Additional file 1: Appendix A.

The intervention program involved online messages, online instant responses, a picture-based food log, an audio e-book, and a loyalty e-card. These five aspects are detailed in the following text.

#### Provision of online messages

Twelve themes, one for each week, were planned in advance, and two to three health text or video messages were provided weekly. The message content included the five concepts (i.e., perceived susceptibility, perceived severity, cues to action, perceived barriers, and self-efficacy) of the HBM as well as simple techniques for implementing healthy eating (Additional file 1: Appendix A.). Messages were designed according to the concepts of knowledge transfer, knowledge clarification, and life application, and were disseminated directly or through a question-and-answer approach. This approach was conducted by the instructors who sent questions via the LINE group which the group members then answered.

Knowledge transfer and life application involved providing information on various meal combinations recommended by the Health Promotion Administration (HPA) of Taiwan, including Taiwanese breakfast, Taiwanese snacks, cafeteria food, and convenience store meals. Examples of a direct message are as follows: (1) “A healthy day starts with breakfast. Do you know what type of breakfast is the healthiest? The figure below provides an example.” and (2) “Let’s see whether you still remember ‘My Healthy Eating Plate.’ Click on the link below to refresh your memory.” Examples of question-and-answer content are as follows: (1) “What type of food will make you sleepy while driving?” (2) “Do you know that your eyes need protection while driving under the sun for a long time? Do you want to know which vegetables and fruits can protect your eyes?” and (3) “Do you know what a ‘sweet burden’ is? Guess how many cubes of sugar someone who consumes 2,000 cal a day can eat?” The video messages involved precautions for maintaining healthy eating habits and continuous exercise.

#### Instant responses

A licensed dietitian and a trained nurse served as online coaches to provide instant responses and cues to action and reduce perceived barriers. This study provided online coaching services from 8 am to 8 pm daily, including weekend. When participants sent messages or asked questions individually, coaches would provide an instant response for immediately solving the participants’ diet-related problems.

#### Audio e-book

Because it is inconvenient and dangerous for truck drivers to read while driving, we converted the health manual into an audio e-book. This step increased the possibility of exposing the truck drivers to health information and improved accessibility to cues to action.

#### Picture-based food log

Participants captured photos of their meals and snacks and uploaded them to the LINE group. The online coaches provided suggestions for improvement or encouragement according to My Plate established by the Ministry of Health and Welfare of Taiwan in 2019.

#### Loyalty e-card

We designed a point accumulation mechanism using a loyalty e-card to encourage drivers in the social media group to participate in online activities. Under the incentive mechanism, the drivers were awarded one point for uploading a photo of one meal in the first 4 weeks. From weeks 5 to 12, the participants were required to upload two photos of their meals to receive one point. In week 12, one additional point was awarded if the drivers’ meals met the criteria (types and servings of food) of My Plate. One point was awarded each time a participant answered an online question correctly. Finally, at the end of this research, the drivers who finished first, second, and third in terms of points at each branch of the company were given a backpack worth US$60, a thermos mug worth US$30, and a US$15 gift voucher, respectively.

### Measurements

The questionnaire was developed based on previous HBM related studies [41–43]. Six experts from nutrition and public health specialties evaluated the content of the questionnaire regarding its applicability, correctness, and completeness to ensure its conformity with the research purpose. The content validity index of the overall questionnaire was 0.79. The questionnaire consisted of the following sections: sociodemographic factors, health examination data, healthy eating behavior, self-perceived health status, self-perceived susceptibility, self-perceived severity of chronic diseases, barriers to healthy eating, self-efficacy of healthy eating, and cues to action. We conducted principal component factor analyses for the sections mentioned above, besides sociodemographic factors, health examination data, as well as healthy eating behavior, and retrieved one factor from each section. The cumulative explained variances of the sections were 60.7% for self-perceived health status, 83.7% for self-perceived susceptibility of chronic diseases, 87.3% for self-perceived severity of chronic diseases, 72.4% for barriers to healthy eating, 83.5% for self-efficacy of healthy eating, and 81.8% for cues to action. Thus, we next calculated the total score by averaging the item scores since the items of each section reflect one domain.

#### Self-perceived health status

The measurement of self-perceived health status was developed based on the questions of the Taiwan National Health Interview Survey and consisted of five items [42]. The items included three forward-scored items: “In general, what do you think of your current health condition?” “I think that my current health condition is very good,” and, “I think that I am healthier than other people the same age as me,” and two reverse-scored items: “I think that I get sick more easily than others do” and “I think that my health is becoming worse.” The responses were measured using a 5-point scale ranging from 1 (strongly disagree) to 5 (strongly agree). The average of the five items created the total score (ranging from 1 to 5) with a higher score indicating a more positive self-perceived health status. The Cronbach’s α value was 0.83.

#### Healthy eating behavior

The drivers’ healthy eating behavior was measured using the food frequency scale [44], which consisted of the intake frequency of nine food items: unrefined grains, vegetables, fruits, beans or bean products, seafood, eggs, meat, nuts, and water. The options included “0–1 days per week,” “2–3 days per week,” “4–5 days per week,” and “6–7 days per week.” Each food frequency was evaluated by the criteria of My Plate which indicated the frequency of meat, water, nuts, vegetables and fruit is almost every day, while the frequency of seafood, eggs, beans, and bean products is at least two to three times a week. For each food, a point was awarded if a participant’s consumption was consistent with the suggestion of My Plate. Otherwise, no point was awarded. The total score for healthy eating behavior ranges from 0 to 9, with a higher average score indicating a more favorable healthy eating behavior.

#### Self-perceived susceptibility

The self-perceived susceptibility scale was revised from Huang et al.’s study [41]. The respondents were asked how likely the following six diseases will happen within the next six months: heart attack, stroke, kidney disease, diabetes, cancer, and other chronic diseases. The scale was measured by a 4-point scale ranging from 1 for “strongly disagree” to 4 for “strongly agree.” The average of the item scores represented the total score with a higher score indicating a higher self-perceived susceptibility. The Cronbach’s α value was 0.96.

#### Self-perceived severity

The self-perceived severity scale was revised from Huang et al.’s study [41]. The respondents were asked how severe they thought the six aforementioned diseases would be if they were diagnosed in the next six months. A 4-point scale ranging from 1 for “not very serious” to 4 for “very serious” was used for assessment. The average of the six item scores was calculated as the total score, with a higher score indicating a higher self-perceived severity. The Cronbach’s α value was 0.97.

#### Perceived barriers

The measurement of perceived barriers was developed according to the previous literature [43, 45] and consisted of two questions: “I don’t like the taste of most high-nutrient foods” and “I think it is difficult to change my eating behaviors to consume more high-nutrient foods within the next two weeks.” These items were scored on a 4-point scale ranging from 1 for “strongly disagree” to 4 for “strongly agree.” The average of the two item scores was used as the total score with a higher score indicating higher perceived barriers to implementing healthy eating behaviors. The Cronbach’s α value was 0.62.

#### Self-efficacy

The measurement of self-efficacy was developed according to the previous research [43, 46, 47]. The scale measured how confident the respondents felt in their ability to manifest healthy eating behaviors in the next two weeks by two items, scored on a 4-point scale. The average of the two item-scores was calculated as the total score for self-efficacy with a higher average score indicating a higher self-efficacy. The Cronbach’s α value was 0.80.

#### Cues to action

Cues to action was measured by a scale which was developed according to previous studies [43, 48]. The scale measured how much the respondents would agree they would follow the information from mass media (e.g., news stories, ads, and other programs), health professionals (e.g., doctors, nurses, and other medical professionals), and laypersons (e.g., family members and friends) about the food choices. The three items were scored on a 4-point scale ranging from 1 for “strongly disagree” to 4 for “strongly agree.” The average of the three item scores was used as the total score of cues to action with a higher average score indicating a higher likelihood to follow cues to action. The Cronbach’s α value was 0.89.

#### Sociodemographic data

The sociodemographic variables considered in this study included birth year, marital status, educational level, and health examination data, including height, weight, waist circumference, high-density lipoprotein, triglycerides, fasting blood glucose, and blood pressure. Information about the variables was provided by the participants on the basis of their annual employee health examination reports. The BMI was calculated from the participants’ height and weight. The metabolic syndrome was determined according to the biochemical value [49].

### Statistical analysis

Statistical analysis was conducted using IBM SPSS Statistics version 19. Means, standard deviations, and percentages were calculated to demonstrate the distributions of sample characteristics. To ensure comparability, we examined the distributions of pretest variables between the experimental and control groups by using the *t*-test and chi-square test. The mean differences between the pretest and post-test results of the two groups were examined by paired *t*-test. Generalized estimating equation (GEE) regression models were adopted to investigate the intervention effects.

## Results

Table 1 presents the distributions of the sociodemographic factors and pretest outcome measures between the intervention and control groups. The majority of the participants had a senior high school degree and 80% of the participants were married. Two in five (44%) had metabolic syndrome, and over half of the drivers were obese. The mean age of the drivers was 47.6 years, and their mean BMI was 27.3 kg/m^2^. Regarding the distribution of the outcome measures, the drivers had low levels of average self-perceived health status and healthy eating scores; moderate levels of self-perceived susceptibility, self-perceived severity, and perceived barriers; and high levels of cues to action and self-efficacy.

**Table 1:** Comparisons of sociodemographic factors and pretest outcome measures of the intervention and control groups

	Total	Intervention group Social media (*n* = 125)	Control group Traditional strategies (*n* = 117)	
	*n* (%)	*n* (%)	*n* (%)	*p* value^a^
**Sociodemographic factors**				
Educational levels				
Low: junior high school or under	56(23.43)	35(28.00)	21(18.42)	0.207
Middle: senior high school	155(64.85)	77(61.60)	78(68.42)	
High: college or above	28(11.72)	13(10.40)	15(13.16)	
Marital status				
Unmarried	33(13.75)	13(10.48)	20(17.24)	0.270
Married	192(80.00)	104(83.87)	88(75.97)	
Divorced	15(6.25)	7(5.65)	8(6.90)	
Metabolic syndrome				
Yes	90(44.55)	44(40.37)	46(49.46)	0.205
No	112(55.45)	65(59.63)	47(50.54)	
BMI category				
Normal	38(18.54)	22(19.64)	16(17.20)	0.729
Overweight	63(30.73)	36(32.14)	27(29.03)	
Obese	104(50.73)	54(48.21)	50(53.76)	
	Mean ± SD	Mean ± SD	Mean ± SD	*p* value^b^
Age	47.6 ± 6.6	47.75 ± 5.41	47.37 ± 7.77	0.020*
BMI	27.3 ± 3.8	27.09 ± 3.96	27.5 ± 3.55	0.776
**Outcome measures**				
Self-perceived health status (range: 1–5)	2.44 ± 0.65	2.36 ± 0.65	2.53 ± 0.65	0.745
Self-perceived susceptibility (range: 1–5)	2.91 ± 0.66	2.91 ± 0.64	2.92 ± 0.68	0.562
Self-perceived severity (range: 1–5)	3.09 ± 0.76	3.10 ± 0.75	3.08 ± 0.77	0.889
Perceived barriers (range: 1–4)	2.57 ± 0.36	2.56 ± 0.37	2.57 ± 0.34	0.663
Cues to action (range: 1–4)	3.25 ± 0.57	3.27 ± 0.62	3.23 ± 0.51	0.294
Self-efficacy (range: 1–4)	2.84 ± 0.66	2.90 ± 0.71	2.77 ± 0.58	0.177
Healthy eating score (range: 0–1)	0.23 ± 0.19	0.22 ± 0.19	0.24 ± 0.18	0.073

^a^ *p* value for the chi-square test

^b^ *p* value for the *t*-test

*BMI* Body mass index

*SD* Standard deviation

No differences existed between the two groups in terms of their educational level, marital status, metabolic syndrome diagnosis, BMI category, and mean BMI distributions (Table 1). However, the mean age of the control group was 0.4 years higher than the intervention group (*p* = 0.02). No differences existed in the seven pretest outcome measures of the two groups. The results indicated good comparability between the intervention and control groups.

Table 2 presents the differences between the pretest and post-test outcome measures of the intervention and control groups. For the intervention group, in the post-test, self-perceived susceptibility, self-efficacy, and healthy eating score increased significantly, and perceived barriers decreased. For the control group, the level of cues to action decreased in the post-test.

**Table 2:** Comparisons of the pretest and post-test outcome measures between the intervention and control groups

	Total (*n* = 242)				Intervention group Social media (*n* = 125)				Control group Traditional strategies (*n* = 117)			
	Pretest	Posttest		Paired *t*-test	Pretest	Posttest		Paired *t*-test	Pretest	Posttest		Paired *t*-test
	Mean ± SD	Mean ± SD	Difference	*p* value	Mean ± SD	Mean ± SD	Difference	*p* value	Mean ± SD	Mean ± SD	Difference	*p* value
Self-perceived health status	2.44 ± 0.65	2.36 ± 0.66	-0.08	0.006	2.36 ± 0.65	2.30 ± 0.68	-0.06	0.194	2.52 ± 0.65	2.43 ± 0.63	-0.09	0.003
Self-perceived susceptibility	2.91 ± 0.66	2.98 ± 0.61	0.07	0.018	2.90 ± 0.64	3.04 ± 0.62	0.14	0.016	2.92 ± 0.68	2.92 ± 0.60	0.00	0.751
Self-perceived severity	3.09 ± 0.76	3.18 ± 0.78	0.09	0.049	3.10 ± 0.75	3.26 ± 0.81	0.16	0.051	3.08 ± 0.77	3.09 ± 0.73	0.01	0.686
Perceived barriers	2.57 ± 0.02	2.40 ± 0.03	-0.17	< 0.001	2.58 ± 0.34	2.27 ± 0.42	-0.30	< 0.001	2.56 ± 0.37	2.54 ± 0.38	-0.02	0.510
Cues to action	3.25 ± 0.57	3.29 ± 0.84	0.03	0.595	3.27 ± 0.62	3.42 ± 1.00	0.15	0.145	3.23 ± 0.51	3.13 ± 0.58	-0.1	0.026
Self-efficacy	2.84 ± 0.66	2.9 ± 0.58	0.08	0.112	2.90 ± 0.71	3.18 ± 0.46	0.28	0.001	2.77 ± 0.58	2.65 ± 0.57	-0.12	0.065
Healthy eating score	0.23 ± 0.19	0.33 ± 0.21	0.10	< 0.001	0.22 ± 0.19	0.39 ± 0.22	0.17	< 0.001	0.24 ± 0.18	0.25 ± 0.16	0.01	0.694

SD standard deviation

The GEE regression results indicated the intervention effects after adjusting the sociodemographic factors (Table 3). The significance of the interaction between time and group indicated, compared with the traditional strategies implemented among the control group, the social-media-based strategies implemented among the experimental group significantly decreased perceived barriers regarding the consumption of a healthy diet (*p* = 0.012), increased willingness to follow cues of action (*p* = 0.036), improved the self-efficacy of healthy eating behaviors (*p* = 0.001), and increased the score of healthy eating behaviors (*p* < 0.001). However, no interaction was observed between time and group when self-perceived health status, self-perceived susceptibility, and self-perceived severity were outcome measures. Overall, the grand mean of self-perceived health status decreased. In addition, educational level was positively associated with self-perceived health status and self-efficacy.

**Table 3:** Results of generalized estimating equation modeling for the outcome measures

Parameter	Self-perceived health status			Self-perceived susceptibility			Self-perceived severity			Perceived barriers			Cues to action			Self-efficacy			Healthy eating score		
	Beta	SE	*p*	Beta	SE	*p*	Beta	SE	*p*	Beta	SE	*p*	Beta	SE	*p*	Beta	SE	*p*	Beta	SE	*p*
**Time**																					
1 = Post-test / 0 = Pretest ^a ^	-0.111	0.038	0.003	0.041	0.025	0.102	-0.017	0.026	0.518	-0.028	0.039	0.474	-0.063	0.048	0.184	-0.067	0.077	0.384	0.016	0.025	0.518
**Group**																					
1 = Intervention / 0 = Control ^b^	-0.143	0.093	0.123	0.019	0.098	0.844	0.030	0.107	0.778	0.052	0.052	0.311	-0.001	0.082	0.992	0.156	0.096	0.104	-0.027	0.025	0.287
Time * Group																					
1 = Post-test / 0 = Pretest * 1 = Intervention / 0 = Control ^c^	0.046	0.064	0.471	0.095	0.060	0.114	0.165	0.089	0.063	-0.282	0.064	< 0.001	0.260	0.124	0.036	0.368	0.113	0.001	0.172	0.037	< 0.001
**Sociodemographic factors**																					
Age	0.003	0.0097	0.786	-0.005	0.010	0.618	0.020	0.010	0.052	0.012	0.005	0.029	0.007	0.007	0.348	0.014	0.007	0.038	-0.003	0.002	0.138
Married																					
2 = Unmarried / 0 = Married	-0.119	0.164	0.469	0.106	0.158	0.502	0.113	0.166	0.498	0.007	0.073	0.923	0.030	0.091	0.741	0.031	0.126	0.806	-0.014	0.034	0.674
1 = Divorced / 0 = Married	-0.223	0.221	0.313	0.346	0.202	0.087	0.327	0.163	0.044	0.105	0.097	0.282	0.059	0.125	0.636	0.059	0.125	0.636	-0.060	0.422	0.156
Education																					
2 = Middle/ 0 = Low	0.234	0.103	0.023	-0.106	1.084	0.328	0.111	0.131	0.397	-0.017	0.056	0.761	0.203	0.084	0.015	0.203	0.084	0.015	0.011	0.023	0.615
1 = High/ 0 = Low	0.364	0.138	0.009	-0.337	0.142	0.017	-0.086	0.143	0.548	0.075	0.088	0.397	0.230	0.115	0.046	0.230	0.084	0.046	0.084	0.039	0.031
BMI	0.009	0.014	0.541	-0.021	0.013	0.111	-0.003	0.014	0.814	0.002	0.007	0.737	0.005	0.010	0.603	0.005	0.010	0.603	0.000	0.003	0.986
Metabolic syndrome 1 = Yes / 0 = No	0.023	0.098	0.817	0.008	0.094	0.939	0.043	0.108	0.692	0.072	0.044	0.102	0.084	0.069	0.280	0.084	0.070	0.228	0.008	0.021	0.692

^a ^Reference group (time): pretest

^b ^Reference group (group): control group

^c ^Reference group (group × time): control group in the pretest

*SE* Standard error

*BMI* Body mass index

In the end, a total of 3,231 points were gathered in the point accumulation activity. On average, each person in the intervention group was awarded 18 points across the 12 weeks, ranging from 5 to 152. With regard to the online instant responses, the sample questions asked by the participants in online instant responses included: “What is the basal metabolic rate?” “What should I do if I feel hungry after eating a piece of bread?” “What is a healthy choice for a midnight snack?” “Does eating half an avocado for breakfast provide enough nutrition?”. Online coaches provided individualized answers for each question. In addition, a total of 1,394 meal photos were uploaded for the picture-based food log. The dietitian gave advisement on healthy food choices according to the uploaded photos.

According to the satisfaction feedback of the participants in the intervention group, 91.2% were satisfied with the overall intervention program. More than 90% of participants believed that the strategies of the intervention program can help implement and maintain their healthy eating behaviors. In addition, 79% of the participants would like to participate in similar activities again in the future.

## Discussion

This study found, compared with a conventional health intervention, a social-media-based intervention for promoting truck drivers’ healthy eating behaviors effectively increased their willingness to comply with cues for action, reduced their perceived barriers, improved their self-efficacy, and increased their healthy eating behaviors. However, the drivers’ self-perceived susceptibility and self-perceived severity of chronic diseases did not change significantly.

A systematic review study indicated most previous intervention studies on truck drivers lacked a control group. Among the studies with a control group, a small number of participants in each group were recruited [50]. The advantage of the research design adopted in this study is the inclusion of an experimental group and a control group with more than 100 truck drivers in each group. Such a design increased the validity of the study.

Truck drivers constrained by working time and place find it difficult to conform to the requirements of conventional intervention programs. If conventional intervention programs (such as courses or manuals) are adopted, a fixed time and location are usually required. This means the number of truck drivers who can participate in such programs is limited [51]. This study used social media to overcome the constraints of time and place, and developed an intervention program to reach more truck drivers. Past studies have indicated the use of social media can facilitate access to groups that are not easily accessible, such as patients with AIDS and firefighters [52, 53], which was verified by this study.

This study referenced past research and developed several online interaction strategies. The first strategy was the provision of regular online information. Previous studies have found providing health columns regularly in newspapers can effectively increase people’s willingness to follow professional recommendations on diet and exercise [54]. Our results echo the previous findings that providing information on a regular basis is a suitable approach for improving the exposure of cues to action. The second strategy involved the provision of instant responses by online coaches. The online coaches in this research provided immediate affirmation and corrections on LINE according to each student’s questions and performance. Studies [55–57] have discovered assistance from health coaches can provide psychological support, reduce unhealthy behaviors of obese individuals, improve weight control, and increase the blood sugar control behaviors of patients with diabetes. The results of this study are consistent with previous studies and indicate having a health coach can enhance the perception of healthy eating among truck drivers. The third strategy adopted in the present study involved using an audio e-book. Studies have noted audiobooks can improve participants’ reading comprehension and enhance the self-efficacy of those who are not interested in reading [58]. Truck drivers do not have time to read when they are on duty. By providing an audio e-book, this study increased the possibility of truck drivers being exposed to cues related to healthy eating. The fourth strategy involved an activity in which the participants uploaded photos of their diet. In this study, photos were used as a replacement for a conventional hard copy diet diary, and these photos enabled the participants to monitor themselves and modify their eating behaviors. Studies have mentioned maintaining conventional hard copy diet records is cumbersome and time-consuming. The use of mobile devices to record diets can reduce barriers to self-monitoring and increase the effectiveness of health interventions [59, 60]. The fifth strategy involved a point accumulation activity based on a loyalty e-card. Studies have discovered a competitive relationship between drivers can contribute to changes in health-related behaviors [15, 61]. In this study, the participants were encouraged to participate in the online point accumulation activity, which facilitated behavior change through competition and incentives.

The results of this study indicated the use of an HBM-based intervention program effectively reduced the participants’ perceived barriers to healthy eating and improved their self-efficacy, compliance with cues to action, and healthy eating behaviors. The findings of this study agree with studies that have used the HBM to develop intervention programs for teenage girls with smoking behaviors, nutritional education programs for patients with diabetes, healthy diets for patients who have undergone cardiovascular operations, and osteoporosis prevention programs for women [38, 62–64].

The present study found overall, regardless of the group, no significant changes occurred in self-perceived health status, self-perceived susceptibility, and self-perceived severity after the intervention. Although a previous study noted significant changes for some of these parameters after a health intervention [37], another study found the these parameters did not exhibit significant changes after a health intervention [34].The results of this study are the same as those of Abood et al. The reasons for these insignificant results might derive from the ceiling effect of these outcome variables or other confounders such as optimistic bias [65].

The advantages of the present study are: First, a social-media-based intervention was used in this study to overcome the time and space constraints caused by the unique working environment of truck drivers. Second, the experimental and control groups contained more than 100 participants each, which increased the statistical validity of the results. Third, various online intervention strategies, including online coaches, instant responses, and an audio e-book, were used to provide the participants with suggestions and supports. Fourth, a point accumulation activity was implemented to establish a competition and incentive mechanism, which enhanced the truck drivers’ motivation to improve their eating behaviors.

The limitations of this study included: First, the physiological indicators of this study were obtained from the participants’ self-reported questionnaires and may have exhibited memory bias. Future studies may directly use the results of health examinations. Second, because the online interaction in this study was conducted through social media, the more active users may have had a higher response frequency in the online interactive community. This might have resulted in uneven intervention. Third, this study only recruited drivers employed by a Taiwanese freight company. Therefore, the results of this study should be generalized with caution. Fourth, the experimental and control groups in this study were from the same company, which might have led to experimental contamination. The significant changes of some outcome variables indicated the effects of intervention were reliable when the possibility of contamination existed. Fifth, this study did not involve a double-blind procedure, so the effect of the intervention might have been affected by the Hawthorne effect. Six, this study did not monitor a delay effect. The sustainability of the project’s impacts therefore cannot be guaranteed. Seven, due to the constraints of workforce and time, the research team did not record the change in food composition consumed by the participants. Future studies can collect participants’ diet diaries to gather more precise data for presenting the effect of the intervention on healthy food consumption.

## Conclusion

This study used LINE as the social media to conduct the intervention programs, which involved online interaction strategies including the provision of online messages, instant online responses, and an audio e-book; a picture-based food log; and point accumulation using a loyalty e-card. The results of this study indicated these strategies can effectively increase the exposure of cues to action, reduce their self-perceived barriers to healthy eating, and enhance their self-efficacy and healthy eating behaviors. The findings of this study can provide health promotion workers with a reference for the development of intervention programs involving diverse methods, such as the use of communication media, to improve the healthy behaviors of truck drivers.

## Supplementary Information

**Additional file 1: Appendix A**. Strategies, learning objectives, digital educational materials, and outcomes of social-media group.

## Data Availability

The datasets generated and analyzed during the current study are not publicly available due to the mandated data permission by the Institutional Review Board of Cathay General Hospital but are available from the corresponding author on reasonable request.
