# Supplementary material for: Quasi-experimental design for using an interactive social media intervention program to improve truck drivers’ health beliefs and eating behaviors
Source: BMC Public Health. 2022 Aug 4;22:1486. doi: 10.1186/s12889-022-13883-6 (PMC9354337; doi:10.1186/s12889-022-13883-6)
Supplement: Supplementary file 1 — Additional file 1: Appendix A. Strategies, learning objectives, digital educational materials, and outcomes of social-media group. [file 12889_2022_13883_MOESM1_ESM.docx]

Appendix A. Strategies, learning objectives, digital educational materials, and outcomes of social-media group

| Strategies | Learning objectives | Digital educational materials | Outcomes |
| --- | --- | --- | --- |
| **Provision of online messages**  Questions regarding increasing the benefits and reducing the barriers of healthy eating behaviors are transmitted by a built-in robot of Line APP. The robot sends correct answers and related health information back to the participants after they reply to the questions. Videos and graphs regarding “My plate” and examples of healthy meals are transmitted by a research assistant manually. | Recognize the benefit of whole grains | Transmit the knowledge about whole grains via sending questions such as:   - “What type of whole grains rich in B complex could reduce fatigue?” - “What kinds of whole grains will give you energy and antioxidants?” - “What kinds of food are considered as whole grain?” | - Increase perceived benefits of healthy foods |
|  | Recognize the benefit of vegetables and fruits | Transmit the knowledge about vegetables and fruits via sending questions such as:   - “Did you know that your eyes need protection when you’re driving under the sun for a long time? Do you want to know which vegetables and fruits can protect your eyes?” | - Increase perceived benefits of healthy foods |
|  | Recognize the harmful effects of refined sugar on your body | Transmit the knowledge about refined sugar via sending questions such as:   - “What type of food will make you sleepy while driving?” - “Did you know what a ‘sweet burden’ is? Guess how many cubes of sugar a person who has 2,000 calories a day may eat?” | - Increase perceived benefits of healthy foods |
|  | Recognize “My Plate” | 1. Provide “My Plate” information via graphs.  2. Provide the videos of "My Plate" produced by the Taiwan Health Promotion Administration   1. Transmit the knowledge about “My plate” via sending questions such as:  - “How much meats and protein will you need per meal?” - “How much vegetables will you need per meal?” - “How much nuts will you need per day?” | - Increase the compliance of the cues to action - Reducing perceived barriers to consuming healthy foods |
|  | Learn how to prepare healthy meals | Provide graphs showing recommendations of healthy meals, including Taiwanese breakfast, Western breakfast, Taiwanese snacks, convenience store meals, cafeteria foods, and Bentos. | - Reducing perceived barriers to consuming healthy foods - Increase the compliance of the cues to action |
|  | Increase the knowledge of healthy eating behaviors | Transmit the knowledge about healthy eating behavior via sending questions such as:   - “Do you know how many chews per bite are recommended to   reduce stomach uncomfortable?”   - “What would you choose the following drinks for breakfast?” - “What would you choose the following cereal for lunch?” | - Increase self-efficacy |
|  | Increase self-perceived susceptibility of chronic diseases | Transmit the knowledge about the relations between food and chronic disease via sending questions such as:   - “What is the relationship between poor eating habits and chronic disease?” | - Increase self-perceived susceptibility |
| **Instant response**  The online coach answers questions and suggests possible options when the drivers raise issues related to healthy eating on the Line APP. | Establish the confidence of discussing issues related to a healthy diet | The sample questions raised by drivers include:   - “What is the basal metabolic rate?” - “What should I do if I feel hungry after eating a piece of bread?” - “What is the healthy choice for a night meal?” - “Does eating half avocado for breakfast provide enough   nutrition?” | - Reducing perceived barriers to consuming healthy foods - Increase self-efficacy |
| **Audio e-book**  The health information is presented in an audio e-book that allows truck drivers to listen when they are driving. | Learn how to self-check physical status and calculate the daily calorie requirement  Recognize risk factors of chronic disease and understand specific methods to reduce the risk factors | The contents of the audio e-book include: Find My Physical Status, Calculate Daily Calorie Requirement, Understand “Healthy Plate”, Remember The Six Tips of “Healthy Plate”, Eat in This Way, My Health Declaration, etc. | - Increase self-perceived severity - Increase self-perceived susceptibility - Increase the compliance of the cues to action - Increase self-efficacy |
| **Picture-based food log**  Drivers take photos of their meals and upload them to the album of the online group. The online coaches provide suggestions or encouragement according to the “My Plate” standards. | Cultivate healthy eating behaviors | Online coaches provide dietary modification services via Line APP after the drivers upload the photos of their meals. For example, the coaches reply: “it would be even better if white rice could be changed to brown rice!” | - Increase self-efficacy - Increase the compliance of the cues to action - Reduce perceived barriers |
| **Loyalty e-card**  The reward points are used to encourage the drivers if they participate in the online activities. | Conduct healthy eating behaviors | The drivers are awarded one reward point for uploading a photo of their meals daily or answering an online question correctly | - Increase self-efficacy - Increase healthy eating behaviors |
